# Frameshifting preserves key physicochemical properties of proteins

**DOI:** 10.1073/pnas.1911203117

**Published:** 2020-03-03

**Authors:** Lukas Bartonek, Daniel Braun, Bojan Zagrovic

**Affiliations:** ^a^Department of Structural and Computational Biology, Max Perutz Labs, University of Vienna, Vienna A-1030, Austria

**Keywords:** frameshift, hydrophobicity, genetic code, evolution

## Abstract

Frameshifts in protein coding sequences are widely perceived as resulting in either nonfunctional or even deleterious protein products. Indeed, frameshifts typically lead to markedly altered protein sequences and premature stop codons. By analyzing complete proteomes from all three domains of life, we demonstrate that, in contrast, several key physicochemical properties of protein sequences exhibit significant robustness against +1 and −1 frameshifts. In particular, we show that hydrophobicity profiles of many protein sequences remain largely invariant upon frameshifting. For example, over 2,900 human proteins exhibit a Pearson’s correlation coefficient R between the hydrophobicity profiles of the original and the +1-frameshifted variants greater than 0.7, despite an average sequence identity between the two of only 6.5% in this group. We observe a similar effect for protein sequence profiles of affinity for certain nucleobases as well as protein sequence profiles of intrinsic disorder. Finally, analysis of significance and optimality demonstrates that frameshift stability is embedded in the structure of the universal genetic code and may have contributed to shaping it. Our results suggest that frameshifting may be a powerful evolutionary mechanism for creating new proteins with vastly different sequences, yet similar physicochemical properties to the proteins from which they originate.

## Significance

Genetic information stored in DNA is transcribed to messenger RNAs, which are then translated to produce proteins. A frameshift in the reading frame at any stage of this process typically results in a significantly different protein sequence being produced. Here, we show that, nevertheless, several essential properties of many protein sequences, such as their hydrophobicity profiles, remain largely unchanged upon frameshifting. This finding suggests that frameshifting could be an effective evolutionary strategy for generating novel protein sequences, which retain the functionally relevant physicochemical properties of the sequences from which they derive.

Frameshifts in the messenger RNA (mRNA) coding sequences of proteins are typically considered to be unproductive events, which, if unchecked, could result in nonfunctional and sometimes even deleterious protein products (1–4). This notion is mainly based on the dramatic difference between the sequences of wild-type proteins and their frameshifted counterparts. For example, the average sequence identity between wild-type human proteins and proteins obtained by +1 frameshifting their mRNAs is only 6.2% (*SI Appendix*, Fig. S1). Following results like this, it has been widely assumed that frameshifting produces polypeptides that are essentially unrelated to wild-type proteins in terms of their physicochemical properties and suitability to carry out biological function (5–7). Equally importantly, frameshifted mRNAs frequently contain premature stop codons and in eukaryotes are rapidly degraded by the nonsense-mediated decay (NMD) machinery (8). It has even been suggested that the genetic code has been optimized such that the hidden stop codons would prevent extensive out-of-frame gene reading (6, 7, 9). More practically, an introduction of frameshifts coupled to NMD has become a standard strategy for disabling gene expression via CRISPR/CAS9 (10).

On the other hand, it is also known that changes in the reading frame do not necessarily lead to unwanted consequences. For example, there exist several known genes that include frameshifts as compared to related genes in other species (11). Moreover, it has been suggested that frameshifts may result in proteins with novel functions (12, 13). Finally, instances of overlapping genes are well described not only in viruses, but even in human (14, 15). In a related context, it has long been known that similar codons encode amino acids with related physicochemical properties (16–18). Although the impact of this feature of the universal genetic code (UGC) has been well appreciated in the case of point mutations, its influence in the case of frameshifts has only recently been addressed (19–21). In particular, Wang et al. (21) showed that wild-type sequences and their frameshifted variants exhibit higher than expected similarity as captured by several classic substitution matrices in sequence alignment with gaps. Furthermore, Geyer and Madany Mamlouk (19) analyzed a particular physicochemical property of amino acids (i.e., a measure of their partitioning in pyridine/water mixtures) and demonstrated a weak, but significant correlation between frameshifted counterparts at the UGC level. Finally, Wnętrzak et al. (20) showed that UGC may have been optimized in part to lessen the impact of frameshift mutations. Importantly, however, all of these studies analyzed a limited set of amino acid properties or substitution frequencies only and focused primarily on the UGC.

The structure, dynamics, and biological function of proteins are determined by the physicochemical properties of their sequences. For example, sequence hydrophobicity profiles of membrane proteins allow one to accurately identify their transmembrane segments (22), while the hydrophobic/hydrophilic alterations in the sequences of cytosolic proteins are seen as important determinants of their tertiary folds (23). Moreover, nucleobase-affinity sequence profiles of proteins have been suggested to provide relevant information about their RNA-interaction propensity (24–27). Finally, the lack of well-defined tertiary structure in intrinsically disordered proteins is directly encoded in the sequences and their physicochemical properties (28). A fundamental question, therefore, is how frameshifting affects the physicochemical properties of real protein sequences. Here, we have analyzed the complete sets of protein sequences in multiple representative organisms and compared them against their +1 and −1 frameshifted counterparts using over 600 different physicochemical properties of amino acids. We show that several such properties are significantly robust against frameshifting, a finding with potentially far-reaching biological implications.

## Results

### Effect of Frameshifting at the Level of the Genetic Code.

**Fig. 1.**
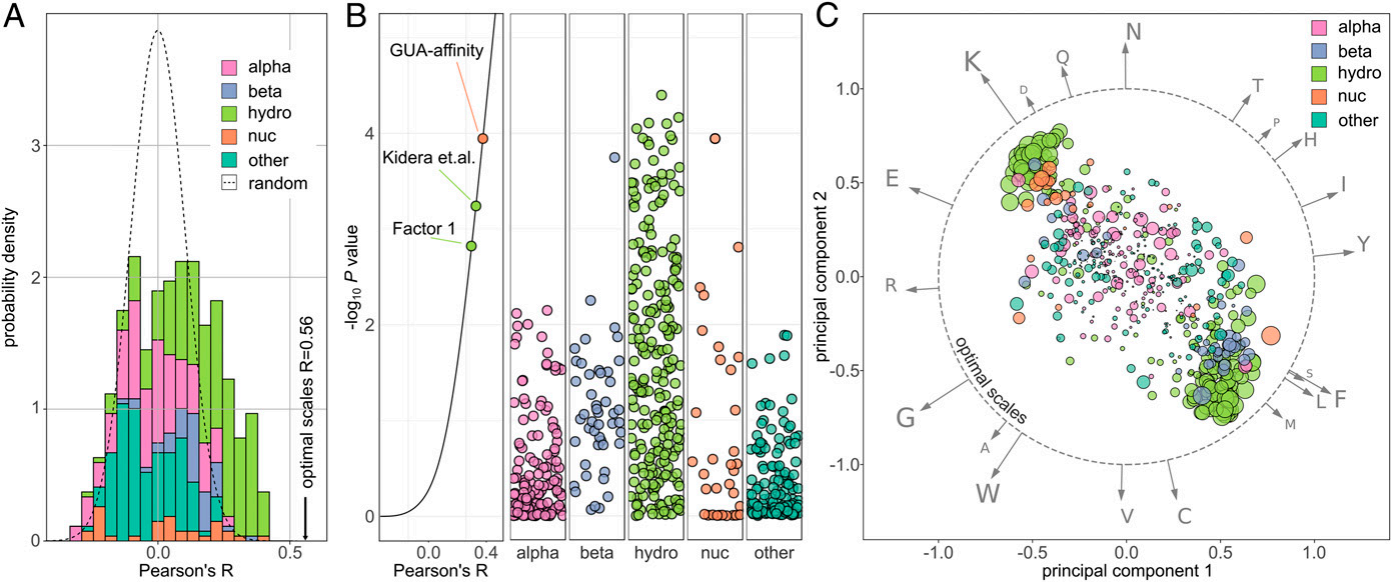
Frameshifting at the level of the universal genetic code (UGC). (*A*) Histogram of Pearson’s correlation coefficients *R* for UGC vs. its frameshifted version for all 604 scales investigated. Scales are grouped by category (alpha: α and turn propensity; beta: β propensity; hydro: hydrophobicity; nuc: nucleobase affinity; other) and presented as a stacked, normalized histogram. The expected density derived via a random model is shown as a dashed line, and the highest achievable Pearson’s *R* obtained for computationally optimized scales is marked by an arrow. (*B*) First panel: Pearson’s Rs for UGC with the associated *P* values, select scales indicated. Other panels: *P* values of 604 studied scales grouped by category with Gaussian jitter added along the *x*-dimension to separate the data points. (*C*) Clustering of scales according to optimal frameshift stability: The 604 studied scales were transformed into the space defined by the PCA of scales computationally optimized for frameshift stability at the UGC level. The first and second principal components of this space (PC1 and PC2) account for almost 100% of explained variance of optimal scales, which therefore lie on a circle (gray, dashed). The 604 physicochemical scales are shown as individual dots whose sizes reflect the negative logarithm of their *P* values as in *B*. Please note how scales with significant frameshift stability tend to cluster in the vicinity of scales with optimal frameshift stability. The arrows correspond to relative contributions of the respective amino acids to PC1 and PC2.

We have first evaluated the impact of frameshifting on different physicochemical properties of individual amino acids as encoded in the UGC. A frameshift of the genetic code table produces a total of 232 pairs involving original and all possible respective frameshifted amino acids (64 × 4 − 24 pairs involving stop codons). As a measure of the impact of frameshifting, we have calculated the Pearson’s *R* over this set of pairs for each of the 604 different amino acid property scales studied (Fig. 1*A*). A distribution of Pearson’s *R*s over all properties corresponds approximately to a Gaussian background distribution calculated from 10^6^ scales with randomly chosen values (see *Materials and Methods* for details). Specifically, the distribution is centered around zero and the best-performing scales reach ∼0.4. In comparison, newly derived scales that were computationally optimized for frameshift robustness in the context of the UGC (see *Materials and Methods* for details) exhibit Pearson’s *R*s of ∼0.56 (Fig. 1*A*, arrow). Importantly, a *P* value analysis demonstrates that a subset of scales, belonging mostly to the hydrophobicity category (Fig. 1, green bars), performs outstandingly well when compared to the random background (Fig. 1*B*).

As representative examples, we highlight two consensus hydrophobicity scales: The Factor 1 scale (*P* = 0.0014), derived by Atchley et al. (29) via factor analysis of more than 500 different amino acid scales, including over 100 hydrophobicity scales, and its predecessor (*P* = 0.0005), derived by Kidera et al. (30) using similar means. A high significance is also reached by several individual scales in other categories, including the knowledge-based scale of amino acid affinity for the RNA/DNA nucleobase guanine (25) and an amino acid β-propensity scale (31). On the other hand, separating the amino acid scales according to physicochemical categories (Fig. 1*B*) reveals the superiority of the hydrophobicity category, with over 100 scales exhibiting *P* < 0.05 (Fig. 1*B* and Datasets S1 and S2). This is supported by an enrichment analysis via Fisher’s exact test showing that hydrophobicity is the only significantly enriched category among scales with *P* < 0.05. Similar results are also obtained with different *P* value cutoffs or a somewhat different assignment of categories (24). Importantly, randomizing the genetic code with its block structure retained yields practically identical *P* values for all scales as the random background used above (*SI Appendix*, Figs. S2 and S3). Moreover, less conservative methods of randomizing the genetic code (e.g., abolishing its block structure) give qualitatively similar results (*SI Appendix*, Figs. S2 and S3). This shows that the significance of frameshift stability of certain physicochemical properties may be intrinsically connected to the specific architecture of the UGC. Finally, our findings are in general agreement with previous studies on the topic (19, 20), although those were limited to just a few specific scales. In contrast, we present a comprehensive analysis of the full scale space providing the necessary context for understanding frameshift stability.

A set of scales that exhibit optimal frameshift stability in the context of the UGC was derived in a consistent manner via different local minimization algorithms (see *Materials and Methods* for details and *SI Appendix*, Fig. S4). This set is practically entirely described by the first two dimensions of a principal component analysis (PCA) and is therefore depicted by the dashed circle in Fig. 1*C*. The relative contributions of individual amino acids to these two PCs are visualized as uniformly rescaled vectors (Fig. 1*C*). A transformation of the 604 studied physicochemical scales to this PCA space reveals strong clustering of hydrophobicity scales near the optimal scales (Fig. 1*C* and *SI Appendix*, Fig. S4). At the same time, scales that themselves exhibit significant frameshift stability, yet belong to other categories, colocalize with the hydrophobicity clusters. This indicates that these scales share certain characteristics that are relevant for frameshift stability with hydrophobicity scales. Please note that the two main clusters shown in Fig. 1*C* are congruent in the sense that inverting a scale moves it to the opposite cluster. In other words, in the case of the hydrophobicity category, the two clusters correspond to hydrophobicity and its inverse, hydrophilicity.

### Effect of Frameshifting at the Level of Protein Sequences.

**Fig. 2.**
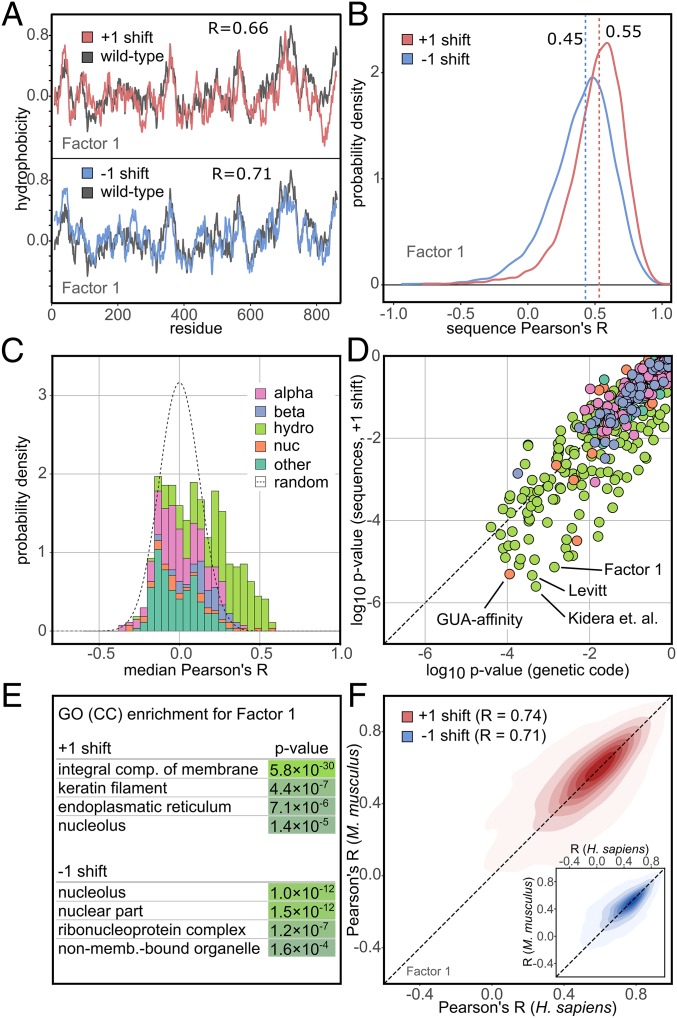
Frameshifting at the level of protein sequences. (*A*) Comparison of wild-type and +1 (*Upper*, red) and −1 (*Lower*, blue) frameshifted Factor 1 hydrophobicity profiles for Ser/Thr phosphatase 4 regulatory subunit 4 protein (UniProtID Q6NUP7) with the associated Pearson’s *R*s. (*B*) Distributions of Pearson’s *R* for wild-type vs. +1 frameshift (red) and wild-type vs. −1 frameshift (blue) for Factor 1 over all human proteins (*n* = 17,083) with their medians indicated. (*C*) Histogram of median Pearson’s *R* for 604 scales when comparing wild-type and +1 frameshifted profiles in human for all investigated scales, grouped by category and presented as a stacked, normalized histogram. The expected density derived via a random model is shown as a dashed line. (*D*) Comparison of *P* values for frameshifting at the level of UGC and +1 frameshifted human sequences for 604 studied scales. (*E*) Enrichment of GO cellular compartment (CC) terms in the top quartile of human sequences according to Pearson’s *R*s between wild-type and +1 or −1 frameshifted Factor 1 profiles (low *P* values: light green; high *P* values: dark green). (*F*) Comparison of Pearson’s *R*s (wild-type vs. +1 frameshifted Factor 1 profiles) between orthologous proteins in *H*. *sapiens* and *M. musculus* (*n* = 12,174; *R* = 0.74). The same is shown for −1 frameshifts in the *Inset* (*n* = 12,174; *R* = 0.71).

In a biological setting, frameshifts always appear in the context of protein sequences. There, the reading frame can be shifted one nucleotide either toward or away from the 3′ end of the mRNA, resulting in two different frameshifted protein variants (+1 shift and −1 shift, respectively). In Fig. 2*A*, we show the hydrophobicity profile of an exemplary wild-type protein (UniProt ID Q6NUP7) overlaid with the hydrophobicity profiles of its +1 and −1 frameshifted variants in the case of the Factor 1 consensus hydrophobicity scale (29). In order to quantify the similarity between the profiles before and after frameshifting, we use the Pearson’s *R*, which in this case is ∼0.7 for both the +1 and the −1 shift as compared to the wild-type (Fig. 2*A*). Importantly, over 2,900 human proteins exhibit a Pearson’s *R* between their wild-type and +1 frameshifted Factor 1 profiles of 0.7 or greater, despite a sequence identity of only 6.5% on average (*SI Appendix*, Fig. S1). In order to illustrate the impact of frameshifts at a proteomic scale, in Fig. 2*B* we show the distributions of Pearson’s *R* as obtained by comparing wild-type Factor 1 profiles against the respective +1 (*R*_median_ = 0.55) or −1 shifted (*R*_median_ = 0.45) profiles over the whole human proteome.

Extending the proteomic analysis to all scales, in Fig. 2*C* we report the distribution over all 604 studied scales of median Pearson’s *R*s in human (wild-type vs. +1 shift) together with the appropriate Gaussian background calculated from 10^5^ scales with randomly chosen values (see *Materials and Methods* for details). As expected from the results obtained at the UGC level, hydrophobicity scales dominate among the scales with the highest median Pearson’s *R*s and the lowest corresponding *P* values in *Homo sapiens* (Fig. 2 *C* and *D*) as well as *Escherichia coli* and *Methanocaldococcus jannaschii* (*SI Appendix*, Figs. S5 and S6). Remarkably, the Factor 1 (29) and Kidera et al. (30) consensus hydrophobicity scales rank among the top four scales in this regard in human, where they are also joined by the Levitt hydrophobicity scale (32) and the knowledge-based scale of amino acid affinity for guanine (25) (Fig. 2*D*). In general, scales that are significantly resistant to frameshifts at the UGC level also exhibit significant invariance in the sequence context. In many cases, in fact, the *P* values tend to be lower in the sequence context than in the case of the UGC (Fig. 2*D*, *SI Appendix*, Fig. S6, and Datasets S1 and S2).

As a next step, we have analyzed the enrichment of different gene ontology (GO) terms for human proteins with the highest frameshift stability using the Factor 1 hydrophobicity scale. For +1 frameshifts, there is a significant enrichment of integral membrane proteins with approximately one-third of all proteins in the top quartile of the human proteome having this GO annotation, while for −1 frameshifts, one observes an enrichment of RNA-binding functions and nucleolus localizations (Fig. 2*E* and Dataset S3). Finally, a comparison of Factor 1 frameshift stability for more than 12,000 orthologous proteins in *H. sapiens* and *Mus musculus* reveals a high degree of similarity (Fig. 2*F*). On the other hand, no such similarity is found for orthologous proteins in organisms belonging to different domains of life (*SI Appendix*, Table S1). Nevertheless, GO analysis in *E. coli* along the same lines as in *H. sapiens* also shows significant enrichment of integral membrane proteins (+1 shift *P* value of 9.7 × 10^−3^).

### Frameshift Stability of a Membrane Protein’s Hydrophobicity Profile.

**Fig. 3.**
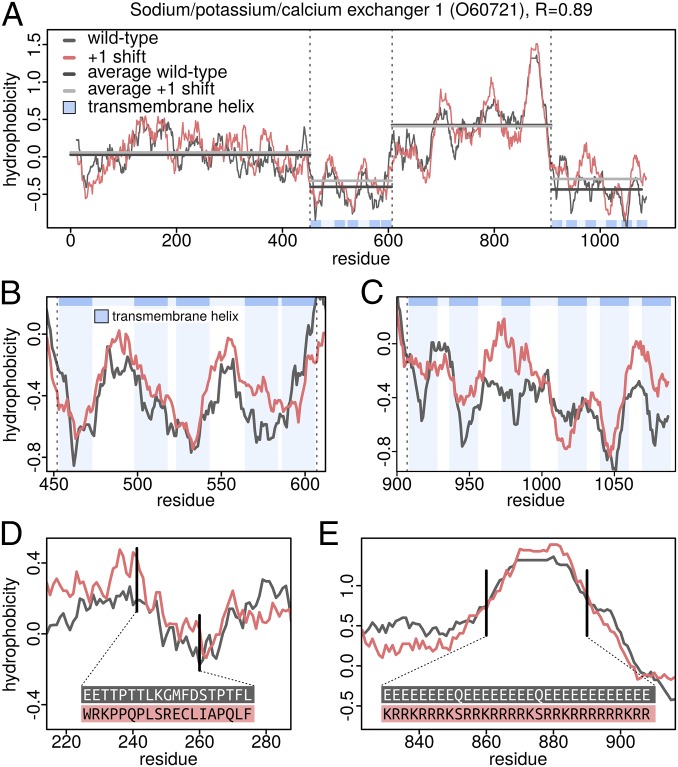
Robustness of a membrane protein’s hydrophobicity profile against frameshifting. (*A*) Factor 1 hydrophobicity profiles of wild-type sodium/potassium/calcium exchanger (UniProtID O60721) and its +1 frameshifted variant with relevant regions indicated with dashed lines. Close-up of the profiles in the first (*B*) and the second (*C*) transmembrane domains of the protein. Note that the specific locations of transmembrane helices are matched in all cases but one. (*D*) Comparison of wild-type and +1 frameshifted sequences in a region outside the transmembrane domains together with the associated Factor 1 profiles. (*E*) Inversion of the charge pattern upon +1 frameshift with a retained hydrophobicity profile.

The strong frameshift stability in the hydrophobicity profiles of many membrane proteins is exemplified in Fig. 3 in the case of sodium/potassium/calcium exchanger 1 (Uniprot ID O60721). The wild-type Factor 1 hydrophobicity profile of this transmembrane protein differs only slightly from its +1 frameshifted variant (*R* = 0.90), despite a sequence identity of 5.4% only. Importantly, different protein domains can easily be identified by analyzing the wild-type Factor 1 hydrophobicity profile with the transmembrane helices adopting extremely low values and the intervening soluble linkers and domains adopting significantly higher values (Fig. 3 *A*–*C*). Remarkably, the +1 frameshifted variant exhibits a pronounced similarity in its alteration of hydrophobic and hydrophilic regions even within the two transmembrane regions (Fig. 3 *B* and *C*). In Fig. 3*D*, we highlight an N-terminal stretch in which only 2 of 19 residues remain the same upon +1 frameshifting and, in Fig. 3*E*, a C-terminal stretch where not a single residue of 31 remains the same, with nevertheless, highly similar hydrophobicity profiles. On the other hand, while the absolute value of electrostatic charge is largely retained upon +1 frameshifting, the shift results in an inversion of the sign of the charge (Fig. 3*E*). In fact, at a whole proteome level, the +1 frameshifting produces sequences whose net charge is negatively correlated with the wild-type net charge (*R* = −0.45), although charge density profiles show a weaker relationship (median *R* = −0.15).

### Frameshift Stability of Nucleobase-Affinity and Intrinsic Disorder.

**Fig. 4.**
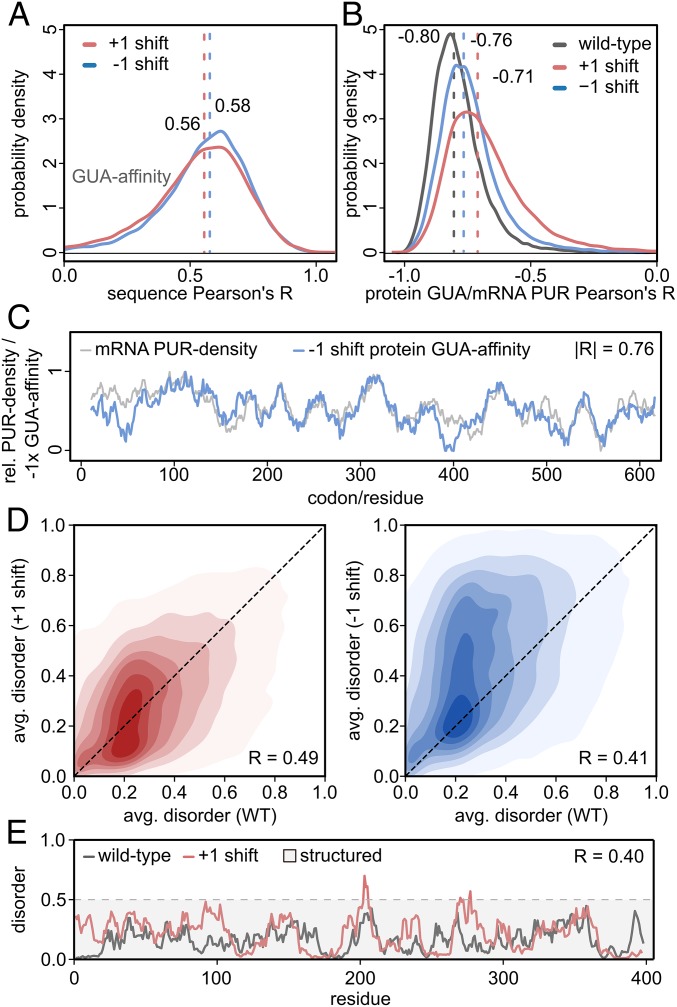
Frameshifting in the context of GUA affinity and intrinsic disorder. (*A*) Distributions of Pearson’s *R* between GUA-affinity profiles of wild-type and +1 or −1 frameshifted human protein sequences (*n* = 17,083). (*B*) RNA vs. protein: Distributions of Pearson’s *R* between mRNA PUR-density profiles and autologous protein’s GUA-affinity profiles in human for wild-type, +1 and −1 frameshifted sequences (*n* = 17,083) with medians indicated. Note that matched profiles are indicated by negative Pearson’s Rs due to the standard definition of GUA-affinity scales. (*C*) RNA vs. protein: Comparison of an mRNA PUR-density profile and protein GUA-affinity profile for the −1 frameshifted sequence of the nuclear RNA export factor (UniProtID: Q9GZY0). (*D*) Comparison of disorder values averaged over full sequences (avg. disorder) in wild-type (WT) and +1 (*Left*, red) or −1 (*Right*, blue) frameshifted sequences. (*E*) Example IUPRED (33) intrinsic disorder profiles of a wild-type protein and its +1 shift variant (UniProtID: P07093).

So far, we have focused mainly on frameshift stability of properties in the hydrophobicity category. As an example of a different scale that also shows significant behavior, we present analogous results for the knowledge-based scale of amino acid affinity for the nucleobase guanine (Fig. 4 *A*–*C*). Importantly, this scale provides context for analyzing frameshift stability from a different perspective: That is, comparing both wild-type and frameshifted protein profiles with the nucleobase density profile of the wild-type coding mRNA.

Namely, we have recently demonstrated a strong matching between nucleobase-density profiles of mRNA coding sequences and the nucleobase-affinity profiles of the proteins they encode (24–27). For example, mRNA purine (PUR) density profiles match their autologous proteins’ guanine (GUA)-affinity profiles with an absolute value of the median Pearson’s *R* of 0.80 in human (wild-type in Fig. 4*B*). We have used this to hypothesize that mRNAs and the proteins they encode could interact in a complementary, coaligned fashion, especially if unstructured (24–27). As indicated above, some nucleobase-affinity profiles of proteins, such as GUA-affinity profiles (Fig. 4*A*) in all studied organisms and adenine (ADE)-affinity profile in *M. jannaschi* (Datasets S1 and S2), exhibit significant robustness against frameshifting. Moreover, the previously observed matching of mRNA purine density profiles and their autologous proteins’ GUA-affinity profiles is also retained for frameshifted proteins, as shown in Fig. 4*B*. Here we show the distributions of Pearson’s *R* between the wild-type mRNA purine density profiles and either wild-type, +1, or −1 frameshifted protein GUA-affinity profiles. As a specific example, the wild-type mRNA PUR-density profile shown in Fig. 4*C* matches the GUA-affinity profile of its −1 frameshifted protein with a Pearson’s *R* of 0.76. This value corresponds to the median of the proteomic distribution (−1 shift in Fig. 4*B*), thus reflecting the high level of profile similarity even in a typical case. This is, in part, a consequence of the robustness in the GUA-affinity profiles (Fig. 4*A*), but even more so a natural corollary of the fact that mRNA nucleobase-density profiles are unaffected by frameshifts combined with the original observation that protein GUA-affinity profiles match their mRNA purine density profiles.

Finally, we have also focused on intrinsic disorder (Fig. 4 *D* and *E*) as a more complex property, which does not only depend on individual amino acids in a given stretch but is also context dependent. The average disorder in wild-type human proteins, as assessed by IUPred (33), correlates with the average disorder of their +1 and −1 frameshifted counterparts with median Pearson’s *R*s of 0.49 and 0.41, respectively (Fig. 4*D* and *SI Appendix*, Fig. S7). Similar results are also seen for sequence profiles of intrinsic disorder. In Fig. 4*E*, we show an example with a Pearson’s *R* of 0.40 between wild-type and +1 frameshift profiles, corresponding approximately to the median of the respective distribution in human. Despite this relatively modest level of correlation in the median case, there still exist thousands of proteins with an undeniably strong frameshift stability. For example, there are close to 2,800 proteins in human for which wild-type disorder profiles correlate with their +1 frameshifted counterparts with a Pearson’s *R* > 0.7 (Dataset S4), with similar results seen for representative organisms from other domains of life (*SI Appendix*, Fig. S8). Finally, it should also be emphasized that the common classification of structured vs. unstructured regions, as given by an IUPRED cutoff of 0.5, is largely retained across the whole exemplary sequence in Fig. 4*E* and, even more importantly, across entire proteomes for average disorder (Fig. 4*D* and *SI Appendix*, Fig. S7).

## Discussion

**Fig. 5.**
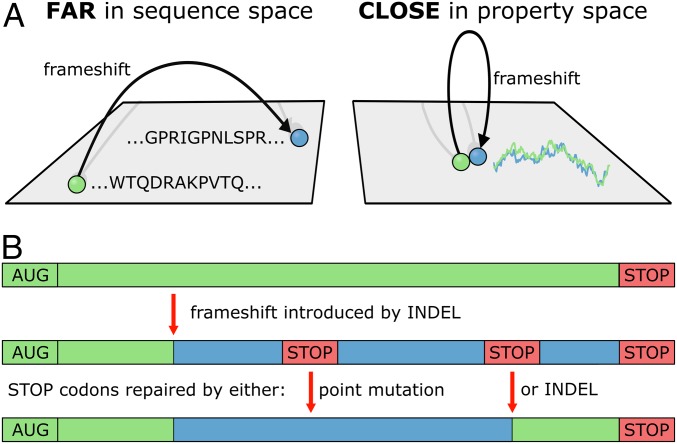
Evolution of protein sequences via frameshifting. (*A*) Frameshifts enable major jumps in protein sequence space with little change in key physicochemical properties like hydrophobicity. (*B*) An insertion or deletion (INDEL) results in a frameshifted gene with potential premature stop codons. These can be removed by either single point mutations or another INDEL-induced frameshift. AUG: start codon.

While frameshift events usually result in markedly altered protein sequences, our findings show that key physicochemical properties of the original sequences are retained in many cases. This immediately suggests a plausible novel mechanism for the evolution of protein sequences: Frameshift mutations could enable major jumps in protein sequence space, while at the same time ensuring that some of the already optimized physicochemical properties of the original sequences are preserved (Figs. 3 and 5*A*). For example, the hydrophobic/hydrophilic sequence patterns are seen as a key feature of proteins when it comes to determining the nature of their three-dimensional structures. By keeping the hydrophobicity profile highly similar, the frameshifted sequence increases its chances of being able to adopt a well-defined fold. Recently, Gardner and colleagues (34) have observed that the predicted secondary and tertiary structure of proteins is relatively robust against point mutations but, unexpectedly, also against frameshifting insertions and deletions. Our present results provide a potential explanation for this finding as frameshifted protein sequences with largely retained hydrophobicity profiles could lead to similar predicted secondary and tertiary structure features.

In a possible scenario of how new sequences could be generated via frameshifting, a gene is duplicated prior to the frameshift event (Fig. 5*B*). The premature stop codons, which are likely to appear during frameshifting, would have to be mutated out, but this burden could be more than compensated for by having a physicochemically optimized starting point for further evolution. Naturally, one can also envision local frameshifts resulting in hybrid sequences, which are part wild-type, part new, increasing greatly the combinatorial richness of the resulting sequences. In this sense, our results capture the most extreme case (i.e., frameshifts of full-length proteins): Hybrid proteins are expected to be even more similar in physicochemical properties to their wild-type counterparts. Recently, Tripathi and Deem (35) provided evidence suggesting that the retention of physicochemical properties of amino acids upon point mutations improves the exploration of functional nucleotide sequences at intermediate evolutionary time scales. We predict that this may also apply to the much more impactful, sequence-altering instances of frameshifting mutations.

It has already been suggested that compositionally similar codons encode amino acids with similar physicochemical properties (16–18), implying that the UGC may have been optimized for robustness against not only point mutations, but frameshifts as well. However, previous investigations of frameshift stability were performed primarily at the UGC level and only considered few amino acid properties or their indirect correlates, as in the case of substitution matrices (19–21). In contrast, our present results are based on a comprehensive analysis of over 600 different amino-acid properties and report on the impact of frameshifts in the case of biologically realistic sequences in multiple organisms. Importantly, correlations at the UGC level are quite weak (Fig. 1*A*) and the realistic impact of frameshifts can only be gauged in the context of protein sequences (Fig. 2). Our results at this level show that frameshift stability is so strong that it indeed might have biologically relevant repercussions. Finally, we provide quantitative evidence that frameshift stability applies to a whole category of different hydrophobicity scales and not just select examples. We find it particularly indicative that two consensus hydrophobicity scales, derived previously by considering hundreds of individual scales, rank in the very top when it comes to frameshift stability (Fig. 2*D* and Datasets S1 and S2).

Our results suggest that, in addition to hydrophobicity, frameshift stability could apply to several other protein sequence properties, including affinity to some nucleobases and structural disorder. It has been proposed that RNA-binding, a process with a strong dependence on hydrophobic forces, was one of the most important functions of ancient proteins (36, 37) and that the UGC was shaped in response to the physicochemical pressures related to such interactions (27, 38, 39). It is possible that the frameshift invariant properties discussed above all partly reflect protein ability to interact specifically with nucleic acids in an unstructured context. The present results also suggest a generalization of our recent complementarity hypothesis. Namely, we propose that mRNAs bind in a coaligned manner not only their autologous proteins, if unstructured, but also their frameshifted variants (24–27). Finally, we also observe a bias for disorder propensity to be retained after frameshifts.

The present analysis opens up several directions for future work. An important frontier concerns the investigation of more complex protein properties that are directly dependent on their primary structure. For example, to what extent are secondary and tertiary structures of wild-type proteins related to those of their frameshifted variants? On a more practical note, what are potential implications of our results in a biomedical context? Is it possible that frameshift robustness could lead to deleterious gain of function? Finally, can evidence be found that frameshifting has indeed played a relevant role during evolution of real proteins? Future studies should shed light on these exciting questions and possibilities.

## Materials and Methods

Complete annotated proteomes of *M. jannaschii*, *Thermococcus kodakarensis*, *E. coli*, *Pseudomonas aeruginosa, M. musculus*, and *H. sapiens* were obtained from the UniProtKB database (40). The corresponding mRNA coding sequences were downloaded from the European Nucleotide Archive Database (41). Sequences including noncanonical amino acids or nucleotides were not analyzed. The majority of the amino acid property scales studied were extracted from the AAindex database (42, 43), and were complemented by additional consensus scales derived by Atchley et al. (29) and an additional category of recently derived nucleobase affinity scales (44–47). The frameshifted variants of individual protein sequences were generated by removing the first four bases (+1 shift) or the first two bases (resulting in the −1 shift) in their wild-type mRNA coding sequences and translating them using the universal genetic code. Protein sequences were then converted to numerical profiles by exchanging each amino acid with its respective scale value and smoothing using 21-residue windows (24). Premature stop codons in frameshifted variants were excluded from the calculation of the average value in a local window, while the size of the window was reduced by their number, except in the case of disorder profiles. In order to capture the effects of frameshifting, Pearson's correlation coefficient R was used throughout as a measure of similarity. Significance analysis was performed on basis of either randomization of the UGC or scales themselves. The PCA of scale space was performed on a set of scales with computationally optimized frameshift stability (26). Experimentally derived scales were subsequently transformed into this PCA space. Enrichment analysis of GO terms was carried out using Gorilla (48), Panther (49), and REVIGO (50) tools. Orthologous genes were sourced from InParanoid8 (51). The disorder propensity of protein sequences was calculated using IUPred (33). Extended methodological details can be found in *SI Appendix*.

### Data Availability Statement.

All data discussed in the paper are available in the *SI Appendix* and Datasets S1–S4.

## Supplementary Material

Supplementary File

Supplementary File

Supplementary File

Supplementary File

Supplementary File
