# Supplementary material for: Frameshifting preserves key physicochemical properties of proteins
Source: Proc Natl Acad Sci U S A. 2020 Mar 3;117(11):5907–12. doi: 10.1073/pnas.1911203117 (PMC7084103; doi:10.1073/pnas.1911203117)
Supplement: Supplementary File [file pnas.1911203117.sapp.pdf]

PNAS
www.pnas.org

Supplementary Information for

Frameshifting preserves key physicochemical properties of proteins

Lukas Bartonek*, Daniel Braun*, and Bojan Zagrovic

* These authors contributed equally to this manuscript.

Bojan Zagrovic
Email: bojan.zagrovic@univie.ac.at

This PDF file includes:

Extended Materials and Methods
Figures S1 to S8
Table S1
Legends for Datasets S1 to S4
SI References

Other supplementary materials for this manuscript include the following:

Datasets S1 to S4

www.pnas.org/cgi/doi/10.1073/pnas.1911203117

Extended Materials and Methods

Data sets Complete annotated proteomes of *Methanocaldococcus jannaschii*, *Thermococcus kodakarensis*, *Escherichia coli*, *Pseudomonas aeruginosa*, *Mus musculus* and *Homo sapiens* together with their mRNA coding sequences were analyzed. Protein sequences were obtained from the UniProtKB database (1) with the maximal-protein-evidence-level set to 4, including only reviewed Swiss-Prot entries. The mRNA coding sequences for each protein were downloaded from the European Nucleotide Archive Database (2). Sequences including non-canonical amino acids or nucleobases were not analyzed.

Amino-acid scales The majority of the amino-acid property scales studied were extracted from the AAindex database (3, 4), and were complemented by additional consensus scales derived by Atchley and coworkers (5) and a number of recently derived nucleobase/nucleotide-affinity scales (6–9). In the rare cases when individual amino acids were not defined in a given scale, they were treated the same way as stop codons (see below). The full set of scales (excluding nucleobase-affinity scales) was categorized following the procedure by Tomii et al. (10) who grouped the scales available in the AAindex into six meaningful categories: “Alpha and Turn propensity”, “Beta propensity”, “Composition”, “Hydrophobicity”, “Physical and Chemical Properties” and “Other”. Additional scales not included in the original analysis by Tomii et al. were added to the existing categories using the same method, except for nucleobase-affinity scales which were added as an additional category. In our presentation of the data, categories “Composition”, “Physical and Chemical Properties” and “Other” were collapsed into a single category “Other”, since all showed no significant effect upon frameshifting and the differences between the groups were not evident.

Sequence identity Sequence identity describes the fraction of strictly identical amino acids that are located at the same position in two protein sequences over the total number of residues. Specifically, in a wild-type protein sequence and its frameshifted counterpart, this refers to locations in the two sequences defined by the respective wild-type codons and their +1 or -1 frameshifted counterparts in the mRNA coding sequence.

Genetic code analysis For each codon in the genetic code table, four associated frameshift codons were constructed by adding one of the four possible bases to the last two bases of the original codon, thereby creating the +1 frameshift graph, yielding 64×4 pairs of native and respective frameshifted codons. Due to this graph being cyclic, there was no need to separately consider the -1 frameshifts. All pairs containing a stop codon were excluded from the set, resulting in 232 frameshift pairs. Utilizing the universal genetic code (UGC), all codons were translated to their corresponding amino acids. Applying one of the 604 amino acid property scales to both original and frameshifted amino acids produced a set of numerical pairs for which the sample Pearson’s correlation coefficient R was calculated. To compare the resulting 604 correlation coefficients against an appropriate background, we carried out the same procedure with 10^6 scales each containing 20 values randomly chosen between -1 and 1. Based on the standard deviation of this random Gaussian background, Z-scores were calculated for each of the 604 scales, which in turn were used to calculate *P* values from an analytic Gaussian distribution. Quantitatively similar results were obtained by randomizing the genetic code while keeping its box-like nature intact (see *genetic code randomization*).

Genetic code randomization Four different methods of generating a random background were used to assess the significance of our observations. First, instead of randomizing the genetic code, the scales themselves were randomized (*“rand. scales”)* as described in the description of “Genetic code analysis” above. Virtually identical significances for individual scales were obtained via a randomization of UGC, while keeping its block structure intact. Specifically, boxes of codons as found in UGC were assigned to new amino acids at random, while keeping the stop codons in their original positions (*“boxes”*). As a third randomization method, all the assignments of codons to amino acids were shuffled, while retaining the absolute number of codons for each amino acid and also retaining stop codon assignments *(“shuffle”)*. Lastly, a complete randomization was performed whereby amino acids were randomly assigned to codons with the only limitation that each amino acid appears at least once and that stop codon assignments again remain unchanged *(“random”)*. In each of the randomization methods, 10^6 random samples were generated. Only in the “rand. scales” method, an analytical Gaussian

background distribution was used to calculate significance, while in the other three methods the number of background samples with better performance was directly used as *P* value.

Optimization of scales The optimization of scales for frameshift resistance was performed via a zero-temperature Monte-Carlo scheme in analogy to our previously published analysis in a different context (11). Briefly, simulated annealing was performed by scaling the step-size with simulation time. A total of 3000 steps was carried out for each Monte-Carlo run. Scales were initialized with random values. At every step, between 1 and 3 scale values, chosen at random, were perturbed by a small time-dependent offset. If the resulting scale resulted in a higher Pearson's R than the original, the move was accepted and otherwise rejected. This resulted in a set of computationally optimized scales with correlations of up to R=0.558 between the UGC and its frameshifted version.

Principal component analysis The PCA was performed using scikit-learn(12) in Python programming language. The PCA space was fitted on a set of 1000 optimal scales for frameshift robustness calculated via Monte-Carlo optimization (as described in "Optimization of scales" above) with an explained variance of 83.2% in PC1 and 16.8% in PC2. The lengths and the directions of arrows in Fig. 1C in the main manuscript correspond to the relative contributions of the respective amino acids to PC1 and PC2. Other local optimization algorithms implemented in scikit-learn (Nelder-Mead, BFGS, trust-constr) yielded theoretical scales resulting in equivalent components in a PCA. Although the ratio of explained variance between PC1 and PC2 varied according to the specific set of optimal scales derived, their total remained ~100% for all cases. In a next step, we transformed the 604 studied 'realistic' scales into this PCA space of computationally derived scales with optimal frameshift stability.

Sequence frameshift generation The frameshifted variants of individual protein sequences were generated by removing the first four bases (+1 shift) or the first two bases (resulting in the -1 shift) in their wild-type mRNA coding sequences and translating them using the UGC. This approach was chosen so that negative shifts could be performed without the need to have additional genomic information beyond the original coding sequence. After frameshifting, the AUG start codon at the beginning of each sequence was removed from all original wild-type sequences in order to enable comparison between equally long protein sequences. The effect of this on the obtained results is negligible since the first three bases contain identical information in all sequences (AUG in mRNAs, Met in protein sequences).

Profile calculation and comparison Protein sequences were converted to numerical profiles by exchanging each amino acid with its respective scale value for each of the 604 different physicochemical property scales. Protein profiles were subsequently smoothed using a 21-residue window, to reduce noise and highlight global features. Profiles stemming from wild-type and frameshifted protein sequences were compared via the sample Pearson's correlation coefficient R. For each property in question, the full distribution of Pearson's Rs between wild-type and frameshifted profiles of all sequences in a given proteome was evaluated and its median used to assess the associated statistical significance in comparison with a randomized background. Specifically, we have derived the distribution of Pearson's R between wild-type and frameshifted profiles for a given proteome for 10^5 scales each containing 20 values randomly chosen between -1 and 1. The standard deviation of the distribution of median values corresponding to this random Gaussian background was subsequently used to calculate the Z-scores and *P* values for each of the 604 property scales studied. Finally, in one instance, protein profiles were compared against mRNA nucleobase-density profiles (Fig. 4B, 4C in the main manuscript). For this, codons were converted to a number between 0 and 3, representing the number of specific bases they contained and subsequently smoothed using a 21-codon window.

Stop codons In most cases, frameshifted mRNA sequences contain premature stop codons. For the calculation of protein sequence profiles corresponding to different physicochemical properties of frameshifted variants, such positions were excluded from the calculation of the average value in a local smoothing window, while the size of the window was reduced by the number of stop codons in it.

GO term enrichment The analysis of the enrichment of gene ontology (GO) terms in a target set defined as the top quartile of the distribution of Pearson's Rs for Factor 1 frameshifts in *H. sapiens* was done using GOrilla (13) and REVIGO (14) tools. In GOrilla, we used the list of 17083 genes in the *H. sapiens* dataset as the background, for which 16281 GO terms were found. In order to reduce the redundancy of the GOrilla output, each ontology enrichment list - cellular compartment (CC), molecular function (MF) and biological process (BP) - was fed into REVIGO together with the associated multiple-hypothesis corrected *P* values. We used the Lin's measure of similarity with a similarity parameter of 0.5, characterized as a small allowed similarity. In the main text, we present the top four significant hits in the CC ontology for both +1 and -1 frameshifts, while the total REVIGO output of the GO analysis can be found in Supporting Information (Table S3). The same procedure was applied to *M. musculus*. Since organisms from other domains of life are not supported by GOrilla, we used Panther (15) for their GO analysis, with the same parameters whenever possible.

Orthologs Lists of orthologous genes between *M. jannaschii*, *T. kodakarensis*, *E. coli*, *P. aeruginosa*, *M. musculus* and *H. sapiens* were downloaded from InParanoid8 (16) and used to compare Pearson's Rs of proteins' Factor 1 frameshift stability between different organisms.

Intrinsic disorder The disorder propensity of a given protein sequence was calculated using IUPred (17) and the 'long' setting for the size of disorder detection. Disorder profiles obtained from IUPred were smoothed using the same window of 21 residues as in case of other profiles for consistency. Since IUPred cannot handle stop codons, they were accounted for by creating two sequences *in silico*, one with the previous residue replacing the stop codon and one using the following residue. Disorder was calculated for both versions and subsequently averaged.

Data access All relevant original data is provided as Supplementary Material to this publication. In case of already published data, the original publications are cited, and data should be accessed at the original source. All data necessary to replicate our results is accessible.

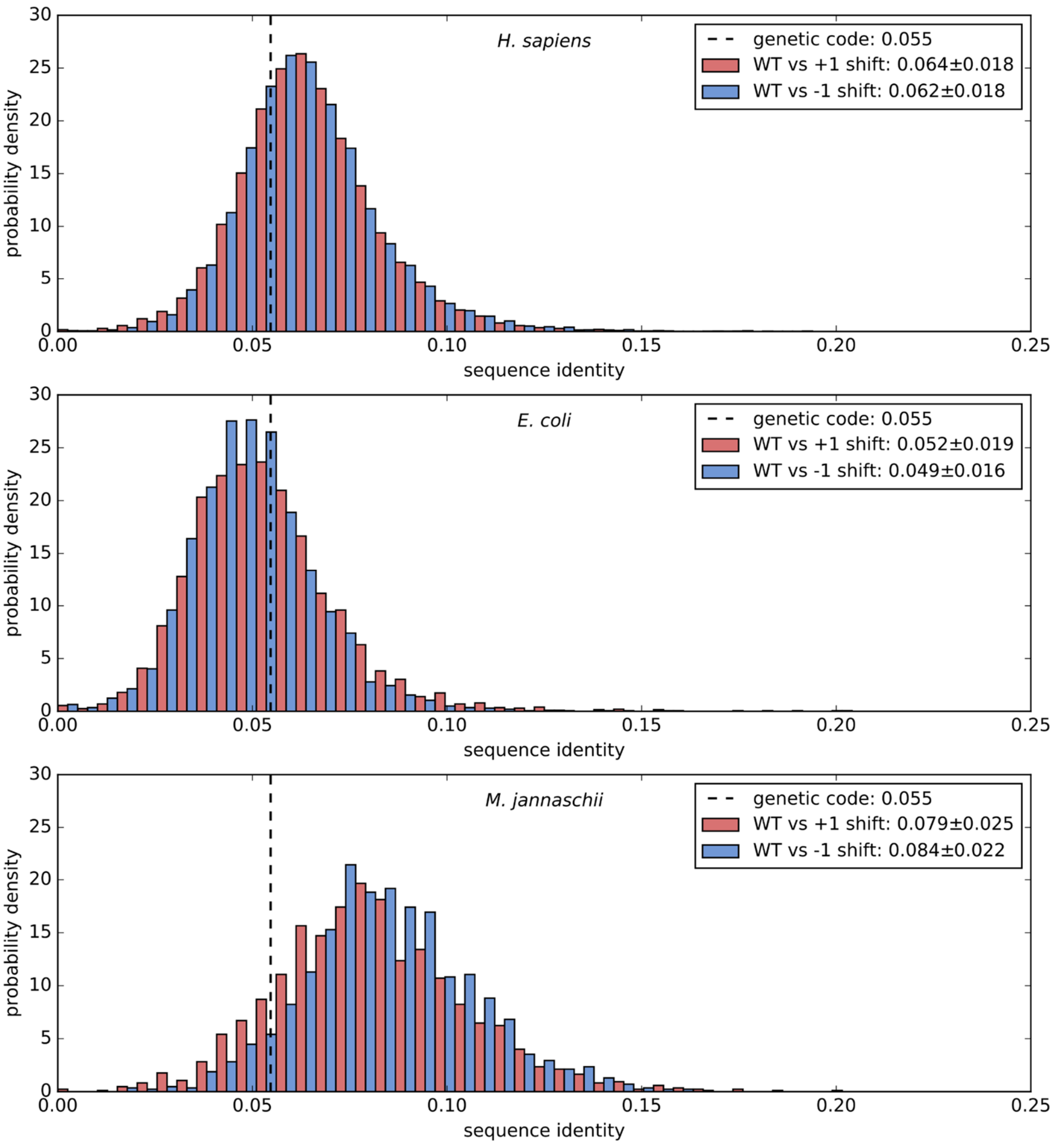

Figure S1. Distributions of sequence identity between wild-type (WT) and frameshifted proteins over complete proteomes of *H. sapiens* (N = 17083), *E. coli* (N = 3939), and *M. jannaschii* (N = 1665). For each distribution, median and standard deviation are shown in the legend. In the universal genetic code, 14 out of 256 frameshift transitions result in the same amino acid, indicated by a dashed line at 5.5%.

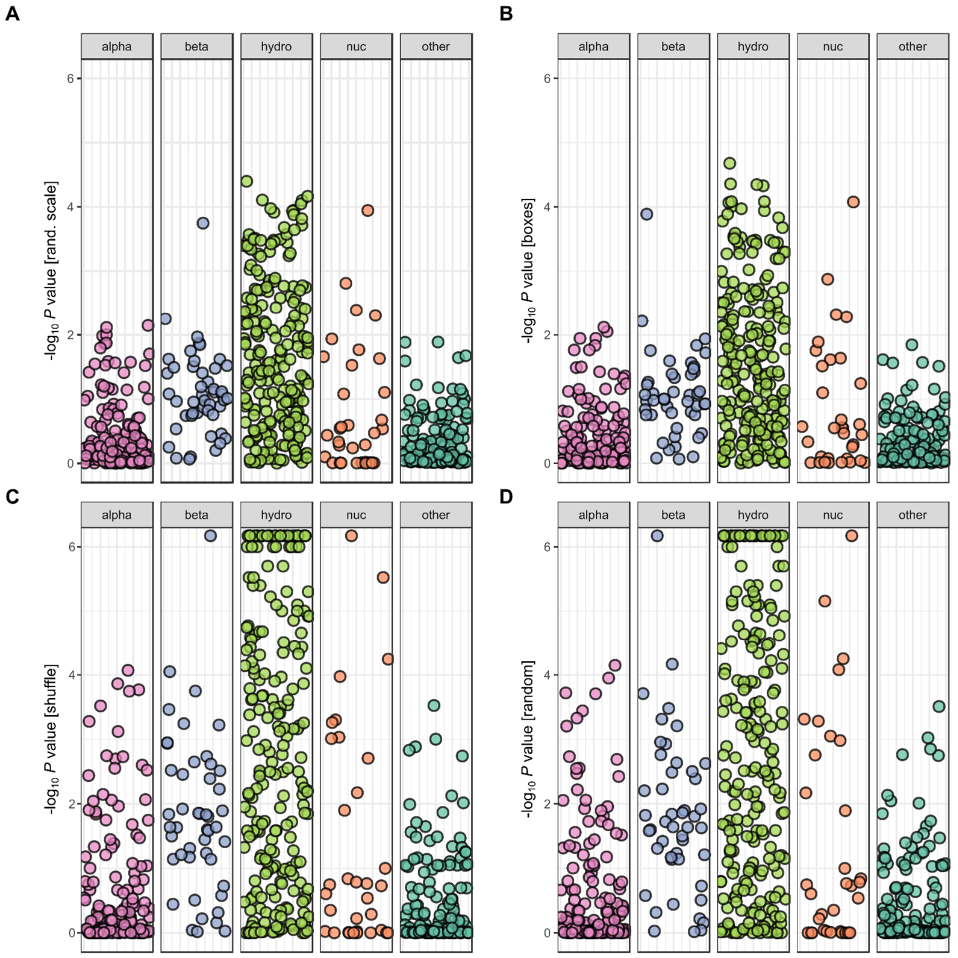

Figure S2. Significance analysis for different randomizations of the universal genetic code (UGC) **A)** *P* values for individual scales as calculated by using a background with randomized scale values. This figure is equivalent to Fig. 1B in the main article. **B)** If the UGC box structure is kept intact while shuffling amino acids in the genetic code, similar results as in **A** are obtained. **C)** By shuffling each amino acid in the genetic code, the box structure of UGC is effectively abolished, resulting in a uniform decrease of *P* values. **D)** Instead of shuffling, amino acids are randomly assigned to codons with the only criterion being that each amino acid is assigned at least once. In all cases, STOP codons were not reassigned during the procedure. It is important to notice that the hydrophobicity category is highly significant regardless of the specific randomization procedure. Scales in panels C and D for which none of the randomized codes performed better than the original are represented with *P* values smaller than 10^{-6}. In all panels, Gaussian jitter is added along the x-dimension with a sole purpose to separate the data points.

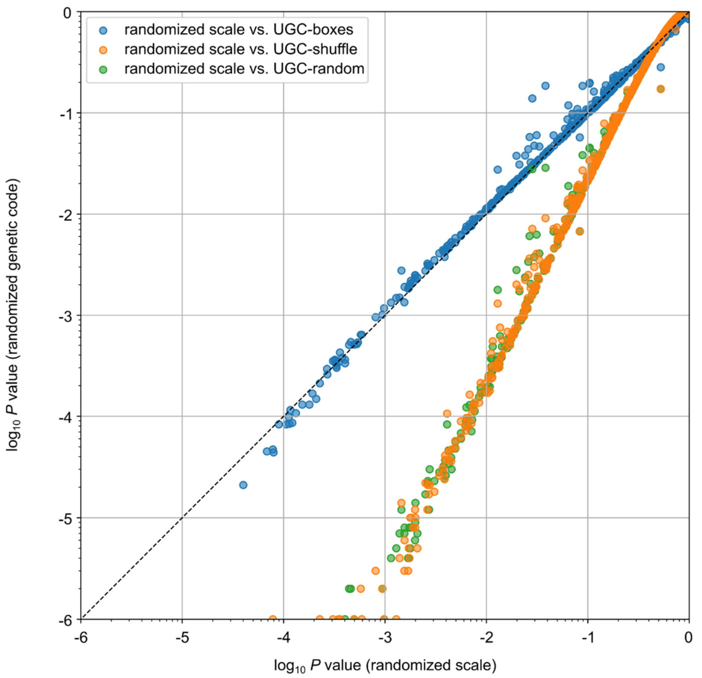

Figure S3. Comparison of different randomization procedures for the generation of the background. Randomizing the universal genetic code while keeping the box structure intact results in quantitatively similar *P* values as using scales with random values as a background (blue). Similarly, the other two methods, shuffling the codon assignments and randomizing the assignments, results in highly similar results (orange and green). Although these two groups differ quantitatively, they still show the same behavior qualitatively.

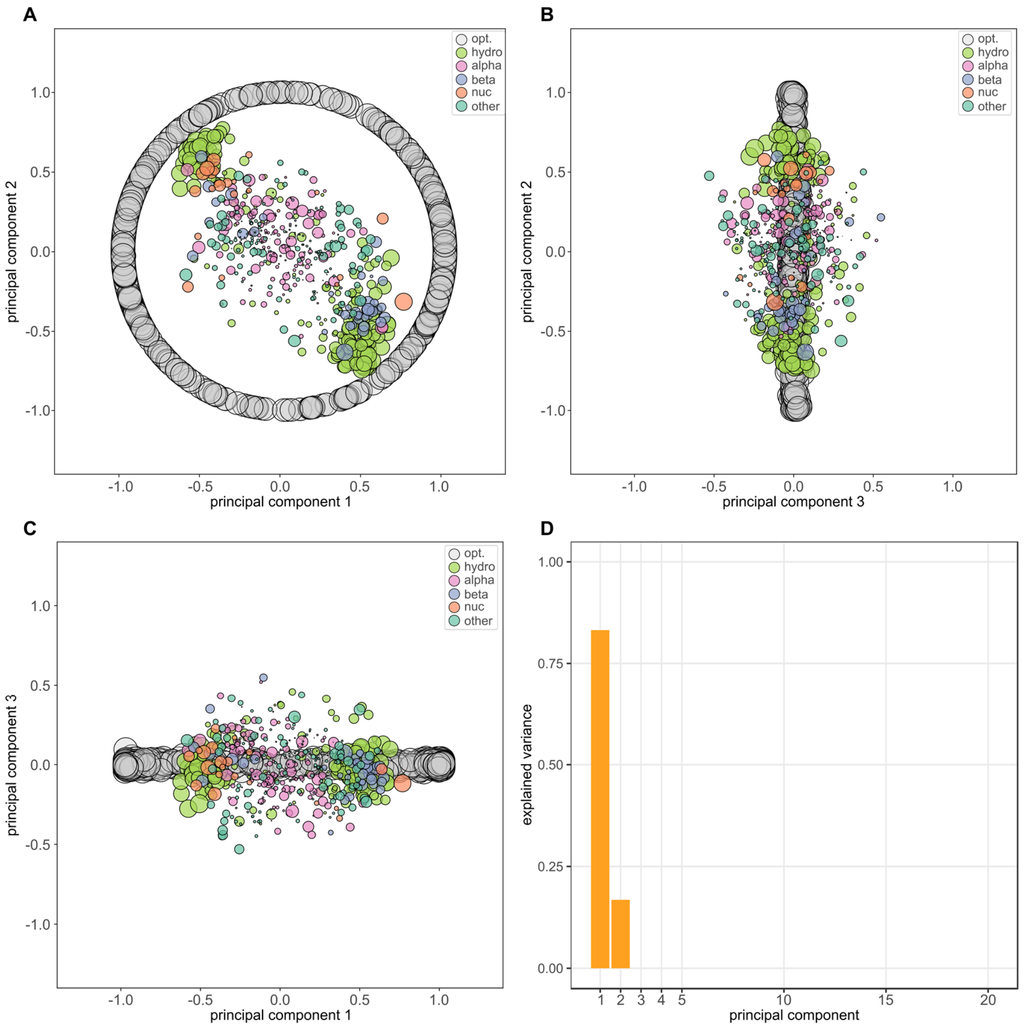

Figure S4. Principal component analysis (PCA) performed on 1000 theoretical scales derived for optimal frameshift stability (grey), together with 604 studied scales (colored according to their category) transformed into this PCA space. Panel **A** is similar to Fig 1C, except that 1000 scales from the Monte-Carlo optimization are explicitly shown. Panels **B** and **C** show PC 3 (0% explained variance of optimal scales) together with either PC 2 (16.8 % explained variance of optimal scales) or PC 1 (83.2 % explained variance of optimal scales). Panel **D** shows the explained variance of the Monte-Carlo optimized scales for each principal component in the PCA.

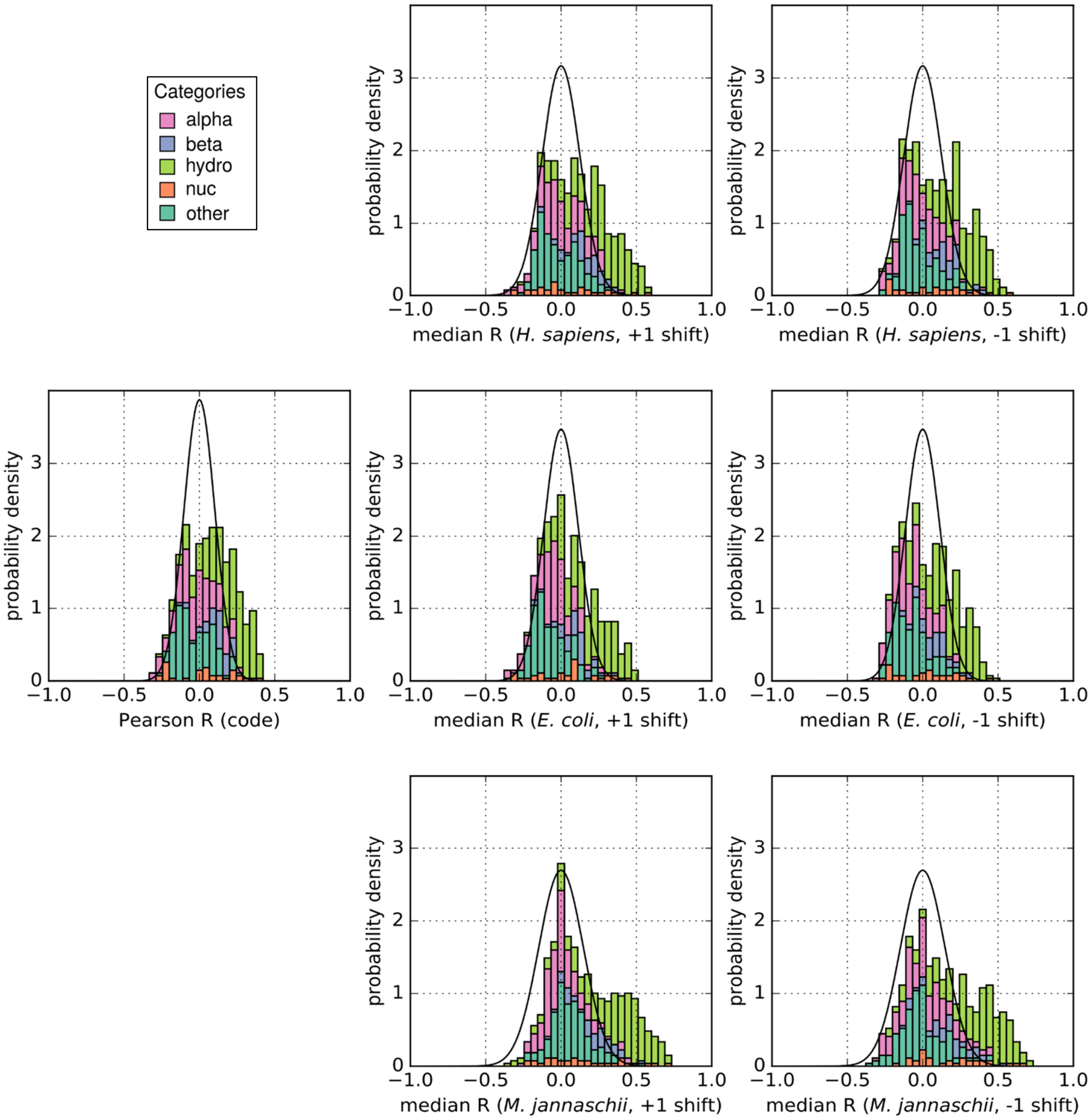

Figure S5. First column: Distribution (stacked histogram) of Pearson's Rs between UGC and its frameshifted variant for 604 different amino-acid property scales grouped by category. The Gaussian curve with a standard deviation of 0.103 corresponds to the random background derived from 10^6 scales with random values. **Second and third column:** Distributions of the median Pearson's Rs over complete proteomes of the indicated organisms for either +1 frameshifted or -1 frameshifted sequences. The random backgrounds were derived from 10^5 scales with random values resulting in standard deviations of 0.126 (*H. sapiens*), 0.115 (*E. coli*), and 0.148 (*M. jannaschii)*. All distributions are normalized and integrate to 1.

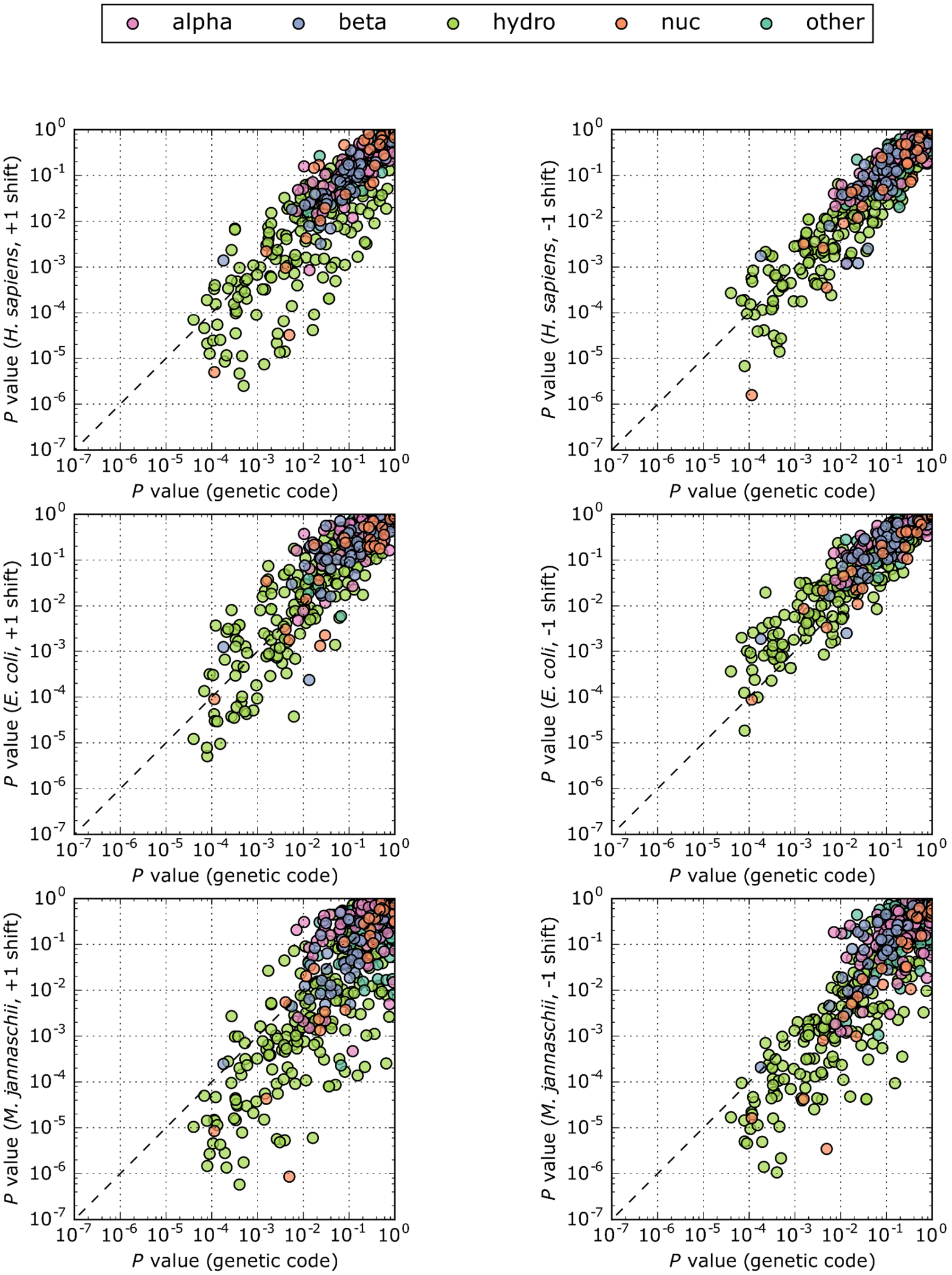

Figure S6. Scatter plots for *P* values of 604 different amino acid property scales in the context of UGC and real biological sequences of *H. sapiens*, *M. jannaschii*, and *E. coli*. The first and second column compare the genetic code with +1 shifts and -1 shifts, respectively.

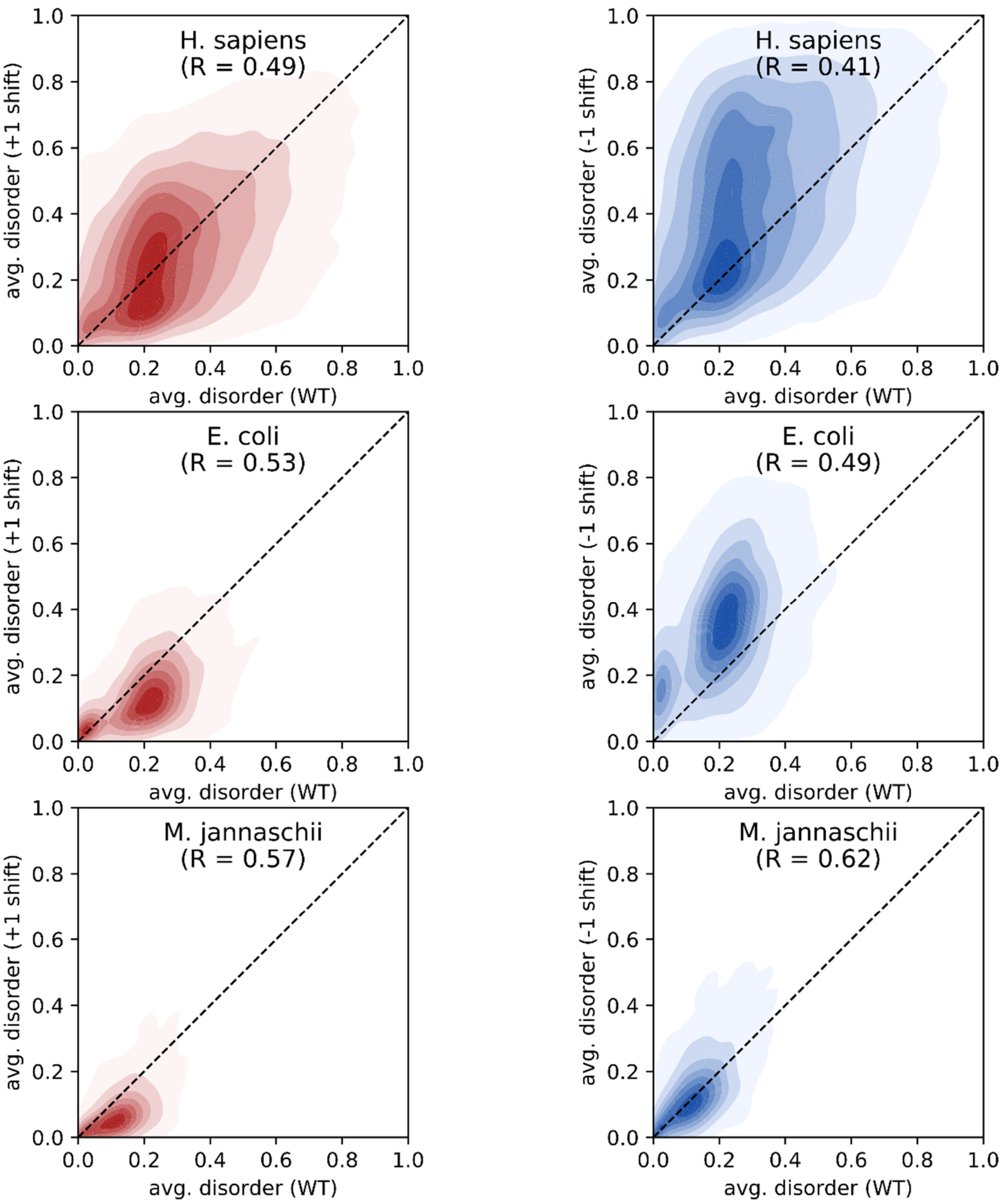

Figure S7. Correlation between the average (avg.) IUPred (17) disorder of wild-type (WT) proteins and +1/ -1 frameshifted proteins over complete proteomes of *H. sapiens* (N = 17083), *E. coli* (N = 3939), and *M. jannaschii* (N = 1665).

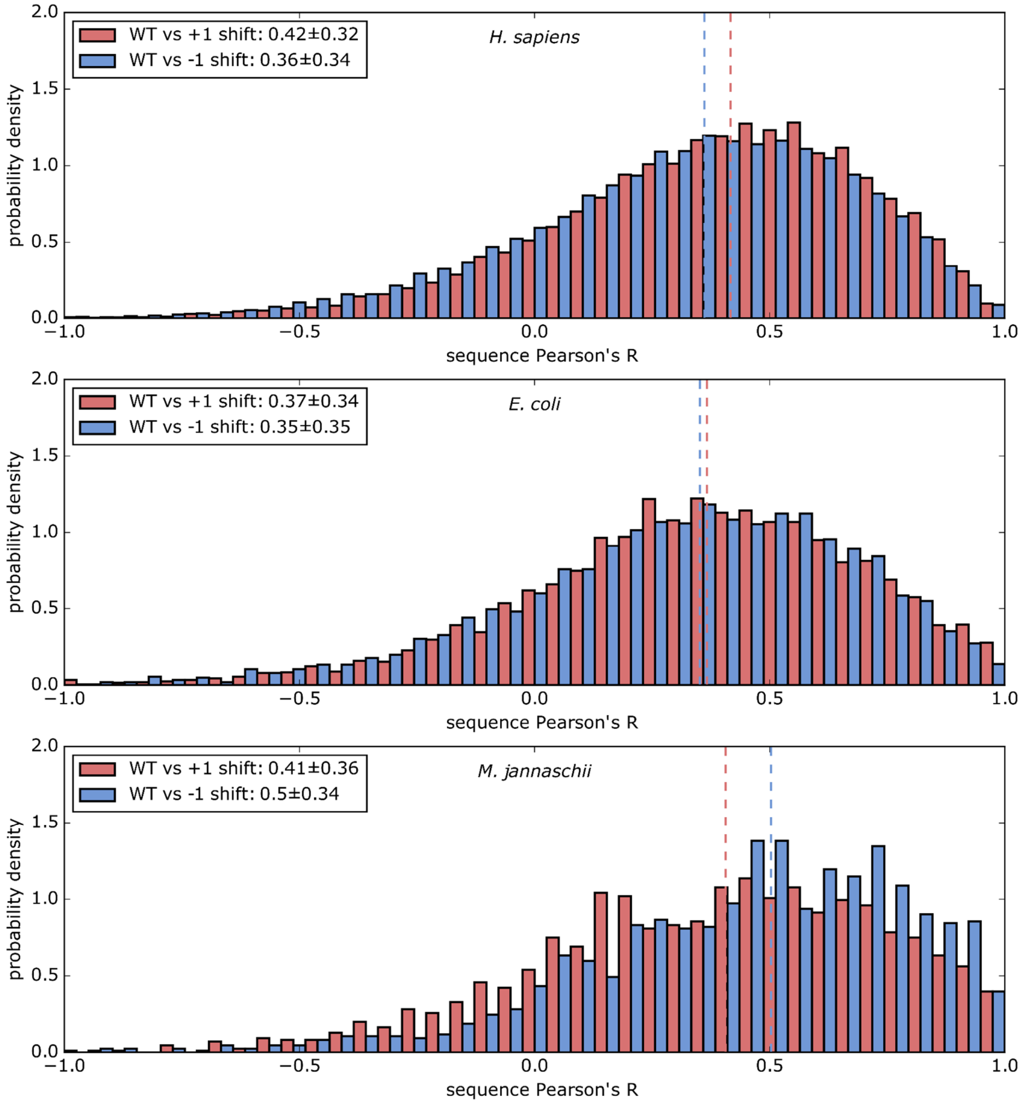

Figure S8. Distributions of Pearson's Rs between intrinsic disorder profiles of wild-type (WT) and frameshifted proteins over complete proteomes of *H. sapiens* (N = 17083), *E. coli* (N = 3939), and *M. jannaschii* (N = 1665). For each distribution, median and standard deviation are shown in the legend, and the median is indicated as dashed line.

Table S1. Comparison of Pearson's Rs (for Factor 1 wild-type vs. frameshifted protein profiles) between orthologous proteins in *H. sapiens, M. jannaschii, E. coli, M. musculus, P. aeruginosa and T. kodakarensis*. The Pearson's R between any two organisms is shown for the +1 shifts and -1 shifts respectively as well as the number of orthologous proteins investigated (N). Higher correlations are observed between more closely related species, with *H. sapiens* and *M. musculus* showing the highest similarities. Very little similarity is observed between different domains of life.

		M. jannaschii	*E. coli*	*M. musculus*	*P. aeruginosa*	*T. kodakarensis*
H. sapiens	*+1 shift*	-0.01	0.10	0.74	0.05	0.14
	-1 shift	-0.06	0.06	0.71	0.06	-0.09
	N	260	485	12174	292	176
M. jannaschii	*+1 shift*	-	0.11	-0.02	0.15	0.20
	-1 shift	-	0.11	-0.04	-0.03	0.07
	N	-	334	237	183	278
E. coli	*+1 shift*	-	-	0.13	0.43	0.24
	-1 shift	-	-	0.12	0.29	-0.04
	N	-	-	448	850	174
M. musculus	*+1 shift*	-	-	-	0.10	0.29
	-1 shift	-	-	-	0.15	-0.03
	N	-	-	-	270	164
P. aeruginosa	*+1 shift*	-	-	-	-	0.33
	-1 shift	-	-	-	-	0.14
	N	-	-	-	-	137

Dataset S1 (separate file). Summary table for 604 different amino acid property scales, including scale categories as defined in this study, Pearson's Rs and *P* values for a frameshift of UGC, as well as medians of Pearson's R over complete proteomes of *H. sapiens* (N = 17083), *E. coli* (N = 3939), and *M. jannaschii* (N = 1665) for either +1 frameshifted or -1 frameshifted sequences.

Dataset S2 (separate file). Summary table with brief descriptions and literature references for all amino-acid property scales listed in Dataset S1. All scales taken from the AAindex (3, 4) are shown together as a set with corresponding literature reference.

Dataset S3 (separate file). Summary table of the output of the gene ontology (GO) analysis for enrichment in the top quartile of the distribution of Pearson's Rs for Factor 1 wild-type/+1 frameshifts and wild-type/-1 frameshifts in *H. sapiens*.

Dataset S4 (separate file). Summary table of selected quantities in +1 and -1 frameshifts of all proteins individually in *H. sapiens, M. jannaschii* and *E. Coli*: sequence identity, Pearson's Rs between wild-type and frameshifted protein sequence profiles using the scales Factor 1 (factor1), Kidera et al. (KIDA850101), GUA-affinity (poly201301) and Levitt (LEVM760101) as well as protein disorder profiles.

SI References

1. T. UniProt Consortium, UniProt: the universal protein knowledgebase. *Nucleic Acids Res.* **46**, 2699–2699 (2018).

2. P. W. Harrison, *et al.*, The European Nucleotide Archive in 2018. *Nucleic Acids Res.* **47**, D84–D88 (2019).

3. S. Kawashima, M. Kanehisa, AAindex: amino acid index database. *Nucleic Acids Res.* **28**, 374 (2000).

4. S. Kawashima, *et al.*, AAindex: amino acid index database, progress report 2008. *Nucleic Acids Res.* **36**, D202–D205 (2007).

5. W. R. Atchley, J. Zhao, A. D. Fernandes, T. Drüke, Solving the protein sequence metric problem. *Proc. Natl. Acad. Sci. U. S. A.* **102**, 6395–400 (2005).

6. M. Hajnic, J. I. Osorio, B. Zagrovic, Computational analysis of amino acids and their sidechain analogs in crowded solutions of RNA nucleobases with implications for the mRNA-protein complementarity hypothesis. *Nucleic Acids Res.* **42**, 12984–12994 (2014).

7. M. Hajnic, J. I. Osorio, B. Zagrovic, Interaction preferences between nucleobase mimetics and amino acids in aqueous solutions. *Phys. Chem. Chem. Phys.* **17**, 21414–21422 (2015).

8. M. Hajnic, A. de Ruiter, A. A. Polyansky, B. Zagrovic, Inosine nucleobase acts as guanine in interactions with protein side chains. *J. Am. Chem. Soc.* **138**, 5519–5522 (2016).

9. C. T. Andrews, B. A. Campbell, A. H. Elcock, Direct comparison of amino acid and salt interactions with double-stranded and single-stranded DNA from explicit-solvent molecular dynamics simulations. *J. Chem. Theory Comput.* **13**, 1794–1811 (2017).

10. K. Tomii, M. Kanehisa, Analysis of amino acid indices and mutation matrices for sequence comparison and structure prediction of proteins. *"Protein Eng. Des. Sel.* **9**, 27–36 (1996).

11. L. Bartonek, B. Zagrovic, mRNA/protein sequence complementarity and its determinants: The impact of affinity scales. *PLOS Comput. Biol.* **13**, e1005648 (2017).

12. F. Pedregosa, *et al.*, Scikit-learn: Machine Learning in Python. *J. Mach. Learn. Res.* **12**, 2825–2830 (2011).

13. E. Eden, R. Navon, I. Steinfeld, D. Lipson, Z. Yakhini, GOrilla: a tool for discovery and visualization of enriched GO terms in ranked gene lists. *BMC Bioinformatics* **10**, 48 (2009).

14. F. Supek, M. Bošnjak, N. Škunca, T. Šmuc, REVIGO summarizes and visualizes long lists of gene ontology terms. *PLoS One* **6**, e21800 (2011).

15. H. Mi, A. Muruganujan, D. Ebert, X. Huang, P. D. Thomas, PANTHER version 14: More genomes, a new PANTHER GO-slim and improvements in enrichment analysis tools. *Nucleic Acids Res.* **47**, D419–D426 (2019).

16. E. L. L. Sonnhammer, G. Östlund, InParanoid 8: Orthology analysis between 273 proteomes, mostly eukaryotic. *Nucleic Acids Res.* **43**, D234–D239 (2015).

17. Z. Dosztányi, V. Csizmók, P. Tompa, I. Simon, The pairwise energy content estimated

from amino acid composition discriminates between folded and intrinsically unstructured proteins. *J. Mol. Biol.* **347**, 827–39 (2005).
